# 105. Fluoroquinolone Stewardship at a Community Health-System: A Decade in Review

**DOI:** 10.1093/ofid/ofab466.307

**Published:** 2021-12-04

**Authors:** Matthew Song, Ashley Wilde, Ashley Wilde, Sarah E Moore, Brian C Bohn, Paul S Schulz

**Affiliations:** Norton Healthcare, Louisville, Kentucky

## Abstract

**Background:**

Fluoroquinolone stewardship is a common target for antimicrobial stewardship programs seeking to maintain or improve fluoroquinolone susceptibility rates. Additional benefits include reducing *C. difficile* infection rates, drug toxicities, and resistance to other antimicrobials as fluoroquinolones can co-select for resistance. The Norton Healthcare antimicrobial stewardship program was founded in 2011 and provides services at 4 adult hospitals with ~1600 beds. Main fluoroquinolone stewardship activities have included provider education, prospective audit and feedback, and guideline and order-set development. The purpose of this study was to describe the resistance and usage rates of fluoroquinolones over time.

**Methods:**

This was a descriptive study examining individual adult hospital antibiograms from 2010 to 2020. Levofloxacin susceptibility rates to *E. coli* and *P. aeruginosa* were collated from annual antibiograms between 2010 and 2020 for outpatients and each adult hospital. Adult hospital resistance rates were aggregated and weighted accordingly to number of isolates per hospital per year. Additionally, levofloxacin and ciprofloxacin inpatient days of therapy (DOT) was collected since 2016 when DOT was first readily retrievable and was normalized per 1000 patient days to compare between different time points.

**Results:**

Outpatient levofloxacin likelihood of activity against *P. aeruginosa* improved from 81% to 91%. Outpatient levofloxacin likelihood of activity against *E. coli* remained stable between 84 – 86% (Figure 1). Adult inpatient fluoroquinolone usage decreased by approximately 75% from 83.5 to 21.37 DOT/1000 patient days since 2016 (Figure 2). Adult inpatient levofloxacin likelihood of activity against *P. aeruginosa* improved from 53% to 83%. Adult inpatient levofloxacin likelihood of activity against *E. coli* improved from 65% to 75% (Figure 3).

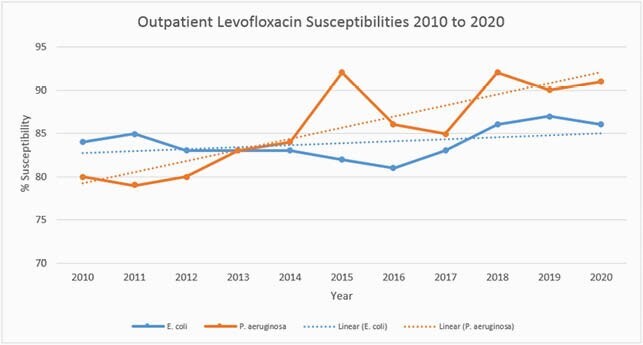

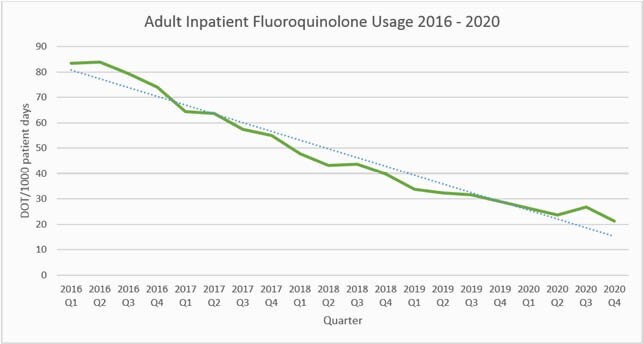

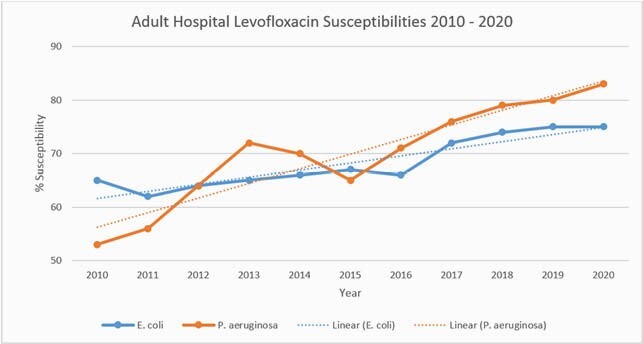

**Conclusion:**

The Norton Healthcare antimicrobial stewardship program has been effective in reducing unnecessary fluoroquinolone usage and improving inpatient fluoroquinolone susceptibility rates. Future studies should examine opportunities to translate successes to the outpatient phase of care.

**Disclosures:**

**Ashley Wilde, PharmD, BCPS-AQ ID**, Nothing to disclose **Paul S. Schulz, MD**, **Gilead** (Consultant, Speaker’s Bureau)**Merck** (Consultant, Speaker’s Bureau)

